# USP19-Mediated Deubiquitination Facilitates the Stabilization of HRD1 Ubiquitin Ligase

**DOI:** 10.3390/ijms17111829

**Published:** 2016-11-02

**Authors:** Kumi Harada, Masako Kato, Nobuhiro Nakamura

**Affiliations:** Department of Life Science and Technology, Tokyo Institute of Technology, 4259-B13 Nagatsuta-cho, Midori-ku, Yokohama 226-8501, Japan; harada.k.ac@m.titech.ac.jp (K.H.); kato.m.ap@m.titech.ac.jp (M.K.)

**Keywords:** deubiquitinating enzyme, endoplasmic reticulum, ERAD, membrane protein, ubiquitin ligase

## Abstract

In the endoplasmic reticulum (ER), misfolded and unfolded proteins are eliminated by a process called ER-associated protein degradation (ERAD) in order to maintain cell homeostasis. In the ERAD pathway, several ER-localized E3 ubiquitin ligases target ERAD substrate proteins for ubiquitination and subsequent proteasomal degradation. However, little is known about how the functions of the ERAD ubiquitin ligases are regulated. Recently, USP19, an ER-anchored deubiquitinating enzyme (DUB), has been suggested to be involved in the regulation of ERAD. In this study, HRD1, an ERAD ubiquitin ligase, is shown to be a novel substrate for USP19. We demonstrate that USP19 rescues HRD1 from proteasomal degradation by deubiquitination of K48-linked ubiquitin chains. In addition, the altered expression of USP19 affects the steady-state levels of HRD1. These results suggest that USP19 regulates the stability of HRD1 and provide insight into the regulatory mechanism of the ERAD ubiquitin ligases.

## 1. Introduction

Ubiquitination, a post-translational modification, regulates a diverse range of cellular functions. It is mediated by the sequential activities of an E1 ubiquitin-activating enzyme, E2 ubiquitin-conjugating enzyme and E3 ubiquitin ligase. Ubiquitination is reversed by deubiquitinating enzymes (DUBs) that cleave off ubiquitin from substrate proteins. Approximately 90 DUBs are encoded in the human genome and classified into six subfamilies, with the largest one being the ubiquitin-specific protease (USP) family [1]. Among the 56 identified USP members, only two (USP19 and USP30) possess the transmembrane (TM) domains. These two are embedded in the endoplasmic reticulum (ER) and mitochondria [2,3]. USP19 also has a soluble isoform lacking the TM domain, while the soluble USP19 has been shown to have an important role in skeletal muscle atrophy [4,5,6,7,8]. Although the precise function of the TM USP19 remains obscure, this USP19 isoform has been shown to be involved in the regulation of the unfolded protein response (UPR), hypoxia response and apoptosis signaling [2,9,10,11]. A previous study reported that TM USP19 is upregulated by ER stress and rescues unfolded/misfolded proteins from ER-associated protein degradation (ERAD) [2], but the role of USP19 in ERAD is controversial [11,12]. Recently, we showed that the TM USP19 regulates the stability and activity of the ERAD ubiquitin ligase membrane-associated RING-CH 6 (MARCH6) via deubiquitination [13]. We therefore hypothesized that TM USP19 may have a role in ERAD and ER protein quality control by regulating the expression and functions of ER-localized ubiquitin ligases. In this study, the ERAD ubiquitin ligase HRD1 was identified as being a substrate protein of TM USP19. It was found that USP19 DUB activity facilitates the modulation of HRD1 stability.

## 2. Results

### 2.1. USP19 Interacts with HRD1

**Figure 1:**
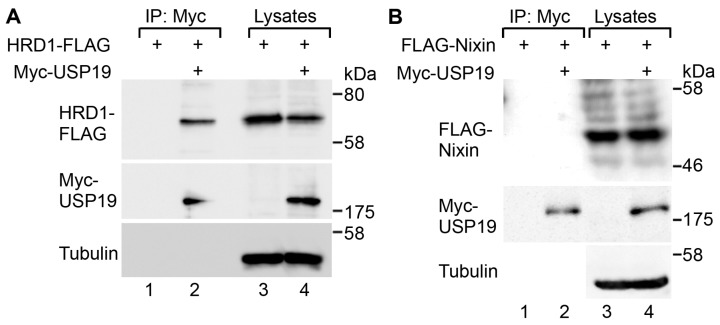
USP19 interacts with HRD1. The 293T cells were transfected with HRD1-FLAG (**A**) or FLAG-Nixin (**B**) along with either a pcDNA3 vector (lanes 1 and 3) or Myc-USP19 (lanes 2 and 4). The cells were lysed and then subjected to immunoprecipitation (IP) with an anti-Myc antibody. The lysates (10% of the input; lanes 1 and 2) and the immunoprecipitates (50% of the eluates; lanes 3 and 4) were analyzed by Western blotting with antibodies against FLAG (**top** panels), Myc (**middle** panels) and α-tubulin (an internal loading control; **bottom** panels).

To assess the possibility that HRD1 is a substrate for TM USP19 (hereafter “USP19”), we first performed co-immunoprecipitation experiments on these proteins. FLAG-tagged HRD1 was transfected along with Myc-tagged USP19 into human embryonic kidney 293T cells. When cell lysates were immunoprecipitated with an anti-Myc antibody, co-immunoprecipitation of HRD1-FLAG with Myc-USP19 was detected (Figure 1A, lane 2). Nixin (ZNRF4) is a recently-characterized ER-bound transmembrane E3 ubiquitin ligase that controls the stability of calnexin [14]. However, there was no association between Myc-USP19 and FLAG-Nixin detected (Figure 1B), supporting the specificity of the USP19 interaction with HRD1. Immunofluorescence microscopy showed that Myc-USP19 was co-localized with HRD1-FLAG, FLAG-Nixin and FLAG-MARCH6 (Figure S1). These results suggest that USP19 interacts with HRD1.

### 2.2. USP19 Deubiquitinates HRD1

**Figure 2:**
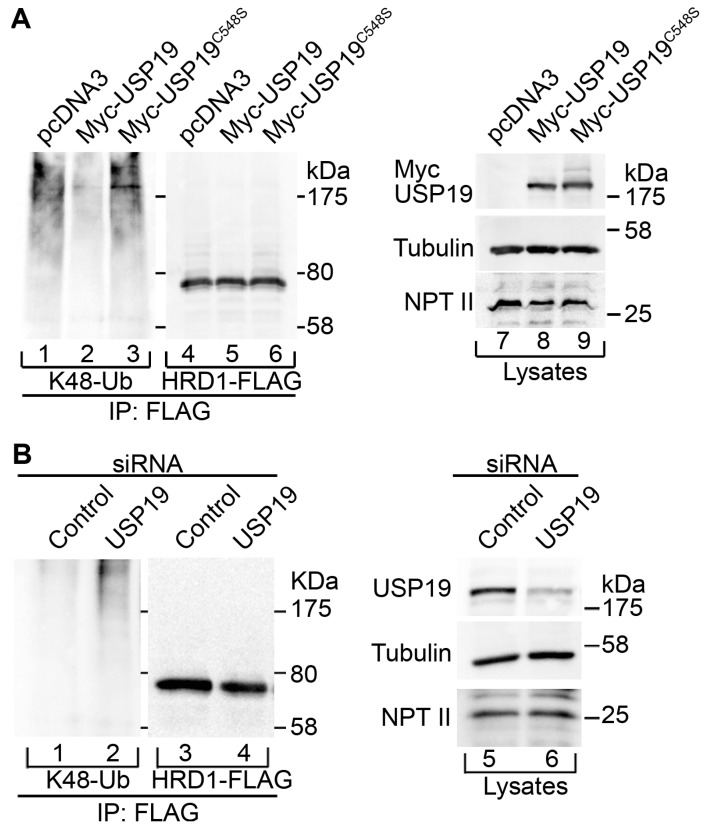
USP19 deubiquitinates HRD1. (**A**) HRD1-FLAG was transfected into 293T cells along with either pcDNA3 (lanes 1, 4 and 7), Myc-USP19 (lanes 2, 5 and 8) or Myc-USP19^C548S^ (lanes 3, 6 and 9). After epoxomicin treatment for 7.5 h, the cells were lysed and then subjected to immunoprecipitation (IP) with an anti-FLAG antibody under denaturing conditions. The immunoprecipitates (50% of the eluates) were analyzed by Western blotting with antibodies against K48-linked ubiquitin (lanes 1–3) and FLAG (lanes 4–6). The lysates (50 μg of protein; lanes 7–9) were analyzed by Western blotting with antibodies against Myc (**top** panel), α-tubulin (**middle** panel) and NPT II (the *Neo*^r^ gene product as a transfection control; **bottom** panel); (**B**) 293T cells were transfected with control siRNA (lanes 1, 3 and 5) or *USP19*-specific siRNA duplexes (lanes 2, 4 and 6). Two days after transfection, the cells were further transfected with HRD1-FLAG. After epoxomicin treatment for 6 h, the cells were lysed and then subjected to immunoprecipitation (IP) with an anti-FLAG antibody under denaturing conditions. The immunoprecipitates (50% of the eluates) were analyzed by Western blotting with antibodies against K48-linked ubiquitin (lanes 1 and 2) and FLAG (lanes 3 and 4). The lysates (50 μg of protein; lanes 5 and 6) were analyzed by Western blotting with antibodies against USP19 (**top** panel), α-tubulin (**middle** panel) and NPT II (**bottom** panel).

The interaction led us to investigate whether USP19 deubiquitinates HRD1. HRD1-FLAG was transfected along with Myc-USP19 into 293T cells. The cells were incubated with the potent proteasome inhibitor epoxomicin for 7.5 h to induce them to accumulate ubiquitinated HRD1-FLAG. HRD1-FLAG was then immunoprecipitated under urea denaturing conditions in the presence of EDTA, *N*-ethylmaleimide and epoxomicin to inhibit post-lysis ubiquitination and deubiquitination. Western blotting of the immunoprecipitates showed that Myc-USP19 overexpression decreased K48-linked ubiquitination of HRD1-FLAG compared to control cells (Figure 2A, lane 1 vs. lane 2; see also Figure S2). However, no such effect was observed when the enzymatically inactive mutant Myc-USP19^C548S^ was expressed (Figure 2A, lane 3). Introduction of a C315S substitution into the catalytic core of HRD1-FLAG resulted in decreased K48-linked ubiquitination, confirming the autoubiquitination activity of this E3 ligase (Figure S3A). The remaining ubiquitination of HRD1^C315S^-FLAG is likely to be mediated by other E3 ligase(s). However, this ubiquitination was not significantly affected by Myc-USP19 overexpression, suggesting that USP19 may preferentially regulate HRD1-mediated autoubiquitination (Figure S3B; compare to Figure 2A). In complementary experiments, siRNA-mediated USP19 knockdown resulted in increased K48-linked ubiquitination of HRD1-FLAG (Figure 2B). No such effect was observed on K48-linked ubiquitination of HRD1^C315S^-FLAG (Figure S3C; compare to Figure 2B). Coomassie blue staining of the cell lysates showed no difference in the protein band pattern between control and USP19 knocked-down cells, suggesting cell viability was not affected by the USP19 knockdown (Figure S4, lanes 4 and 5). In addition, reverse transcriptase (RT)-PCR analysis of *XBP1* mRNA expression showed that ER stress was partially induced, but the level was comparable under these conditions (Figure S5, lanes 4 and 5). These results suggest that USP19 deubiquitinates HRD1.

### 2.3. USP19 Stabilizes HRD1

**Figure 3:**
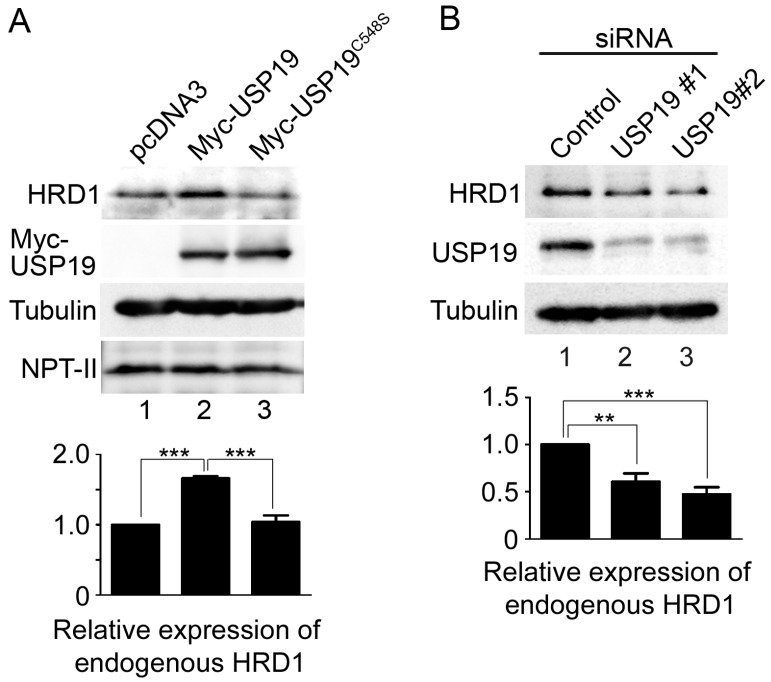
Both overexpression and knockdown of USP19 affect endogenous HRD1 expression. (**A**) The 293T cells were transfected with either a pcDNA3 vector (lane 1), Myc-USP19 (lane 2) or Myc-USP19^C548S^ (lane 3). Whole cell lysates (20 μg of protein) were analyzed by Western blotting with antibodies against HRD1 (**top** panel), Myc (**second top** panel), α-tubulin (**third top** panel) and NPT II (**bottom** panel); (**B**) the 293T cells were transfected with control siRNA (lane 1) or *USP19*-specific siRNA duplexes (lanes 2 and 3). Three days after transfection, whole cell lysates (30 μg of protein) were analyzed by Western blotting with antibodies against HRD1 (**top** panel), USP19 (**middle** panel) and α-tubulin (**bottom** panel). The bar graphs show the relative expression of HRD1 normalized to α-tubulin expression from at least three independent experiments (mean ± SEM). **, *** statistically significant (one-way ANOVA, *post-hoc* test, ** *p* < 0.01 and *** *p* < 0.001, respectively).

**Figure 4:**
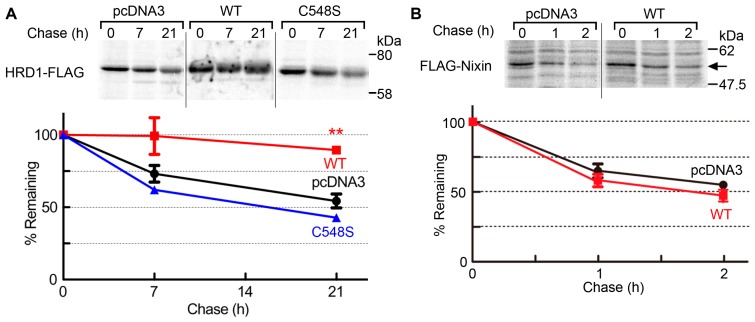
USP19 stabilizes HRD1. HRD1-FLAG (**A**) and FLAG-Nixin (**B**) were transfected into 293T cells along with either a pcDNA3 vector, Myc-USP19 (wild type; WT) or Myc-USP19^C548S^ (C548S). The cells were pulse labeled with ^35^S for 30 min and chased for the indicated periods of time. The cell lysates were immunoprecipitated with an anti-FLAG antibody and then analyzed by SDS-PAGE followed by autoradiography. The data were plotted as a percentage of the remaining proteins relative to time zero from at least three independent experiments (mean ± SEM). ** statistically significant (Student’s *t*-test, ** *p* < 0.01).

We next examined whether the USP19 activity affects the protein expression levels of HRD1 by performing Western blotting on 293T cells transfected with Myc-USP19 or Myc-USP19^C548S^. Myc-USP19 overexpression resulted in a 1.7-fold increase in the levels of endogenous HRD1 (Figure 3A, lane 2), while Myc-USP19^C548S^ had no such effect (Figure 3A, lane 3). Conversely, reduced USP19 expression by siRNA-mediated knockdown resulted in a 0.48- to 0.61-fold decrease in endogenous HRD1 expression (Figure 3B). Under these conditions, cell viability was unaffected and ER stress was not induced by USP19 knockdown (Figures S4 and S5, lanes 1–3). In control experiments, we observed that the altered expression of USP19 had no effect on the expression levels of FLAG-Nixin [15]. Furthermore, we investigated the correlation between USP19 activity and the protein stability of HRD1-FLAG by pulse-chase experiments. As shown in Figure 4A, coexpression of Myc-USP19 resulted in decreased HRD1-FLAG turnover, while Myc-USP19^C548S^ had no or less effect on the stabilization of HRD1-FLAG. Coomassie blue staining of the cell lysates showed no cellular loss under these experimental conditions (Figure S6). In control experiments, we observed that Myc-USP19 did not affect the stabilization of FLAG-Nixin (Figure 4B). We also examined the effect of USP19 activity on endogenous HRD1 stability. The 293T cells transfected with pcDNA3, Myc-USP19 or Myc-USP19^C548S^ were incubated with cycloheximide to block protein translation for 0, 6 and 12 h. Western blotting of whole cell lysates showed that degradation of HRD1 was delayed by the overexpression of Myc-USP19, but not Myc-USP19^C548S^ (Figure S7). These results suggest that USP19 stabilizes HRD1 through its deubiquitinating activity.

In the previous study, we demonstrated that USP19 knockdown results in the increased expression of mutant ABCB11 (ABCB11^G238V^), an ERAD substrate of MARCH6 [13]. We therefore examined whether overexpression and knockdown of USP19 affect the protein expression levels of HMGCR (3-hydroxy-3-methylglutary-coenzyme A reductase), a protein whose basal expression is regulated by HRD1 [16]. As shown in Figure S8A, Myc-USP19 overexpression resulted in increased expression of FLAG-tagged HMGCR, while Myc-USP19^C548S^ had no such effect. In contrast, USP19 knockdown resulted in decreased expression of FLAG-HMGCR (Figure S8B). These results suggest that, in contrast to the case for ABCB11^G238V^, USP19 is likely to stabilize FLAG-HMGCR.

## 3. Discussion

ERAD is one of the most important mechanisms for maintaining cellular homeostasis. It requires four steps, (1) recognition; (2) ubiquitination; (3) dislocation and (4) proteasomal degradation of substrate proteins [17]. Although a number of ER-localized ubiquitin ligases have been identified as responsible for the ubiquitination of ERAD substrates [17], the mechanism underlying the regulation of the ERAD ubiquitin ligases remains to be elucidated. A previous study suggested that TM USP19 acts as a novel ERAD regulator by modulating the stability of MARCH6 ubiquitin ligase [13]. In this study, we showed that USP19 also controls the stability of HRD1 by deubiquitination. These findings support a role for USP19 in the regulation of the ERAD ubiquitin ligases in mammalian cells.

HRD1, a well-characterized ER-resident ubiquitin ligase, targets many unfolded/misfolded proteins to ERAD, thereby preventing cell death due to ER stress [18,19]. Its expression is induced by ER stress at the transcriptional level [18,19]. HRD1 itself is likely to be an ERAD substrate that undergoes proteasomal degradation through autoubiquitination [20]. In the steady state, to avoid premature interruption of protein folding, the activity of the ERAD machinery is probably diminished by their degradation through the proteasomal and lysosomal pathways (ERAD tuning) [21]. Recent studies reported that USP25 counteracts ubiquitination of the ERAD substrates by HRD1 [22,23]. However, the mechanism by which the expression level of HRD1 is regulated remains unclear. We reported that USP19 interacts with and targets HRD1 for the deubiquitination of K48-linked ubiquitin chains, the primary signal for proteasomal degradation [24,25] (Figure 1A and Figure 2A). In this study, we found that overexpression of USP19 also increases the stability and steady-state expression levels of HRD1 in a manner dependent on its DUB activity (Figure 3A, Figure 4A and Figure S7). The opposite effect of USP19 knockdown was observed on the deubiquitination and expression levels of HRD1 (Figure 2B and Figure 3B). Therefore, these results suggest that USP19 positively regulates the HRD1 level by protecting it from ERAD through a DUB activity–dependent mechanism. The activity and function of HRD1 ubiquitin ligase might be modulated in the ERAD system by this regulation of stability. Like HRD1, the expression of USP19 is increased in response to ER stress [2]. USP19 may maintain the stable expression of HRD1 under stressful conditions and thereby afford protection against the cytotoxic effects of misfolded proteins. In the previous study, we have shown that USP19 counteracts the effect of MARCH6 on ABCB11^G238V^ degradation [13]. In this study, however, opposite results were obtained on HMGCR expression (Figure S8). Hassink et al. [2] reported that USP19 interacts with and rescues other HRD1 substrates, cystic fibrosis transmembrane conductance regulator (CFTR) Δ508 and T cell receptor (TCR) α, from ERAD. In a similar way, USP19 might directly protect HMGCR-FLAG from degradation. It cannot be ruled out that USP19 could affect the functions of other E3 ligases involved in HMGCR degradation, such as gp78 and TRC8 [26]. Baldridge and Rapoport [27] reported that, in addition to protein degradation, the autoubiquitination of yeast Hrd1, but not ERAD substrates, is essential for dislocation of ERAD substrates. They have postulated that Hrd1 forms a channel that recognizes ERAD substrates and mediates their translocation across the ER membrane. Polyubiquitination of Hrd1 probably leads to a conformational change in this protein that triggers protein translocation. This raises the possibility that USP19 might negatively regulate HRD1-mediated protein dislocation, thereby leading to increased expression of HRD1 itself. This remains to be clarified in future studies. There are many other ER-localized ubiquitin ligases in mammalian cells [17]. The effect of USP19 on protein stabilization is likely to be specific, because USP19 does not stabilize or interact with another ER ubiquitin ligase, Nixin (Figure 1B and Figure 4B). Further investigation is also needed to determine which ER ubiquitin ligase is a substrate of USP19 and the mechanism underlying substrate specificity.

Recently, Lee et al. [28] reported the role of TM USP19 in misfolding-associated protein secretion (MAPS), a mechanism by which misfolded cytosolic proteins are released into the extracellular environment under a condition of proteasomal impairment. In this activity, USP19 directly recognizes misfolded cytosolic proteins and sorts them into the ER-associated late endosomal vesicles which are destined to fuse with the plasma membrane. USP19-mediated deubiquitination of MAPS substrates promotes their secretion. It is unclear whether substrate ubiquitination is required for MAPS and whether, if so, ERAD ubiquitin ligases are involved in its ubiquitination. In this regard, it is important to clarify whether USP19-mediated stabilization of the ERAD ubiquitin ligases influences the MAPS pathway under stressful and/or disease conditions. Lee et al. [28] also reported that proteasomal inhibition leads to high levels of cell death in CRISPR USP19 knockout cells. However, proteasomal inhibition in combination with USP19 knockdown did not affect cell viability under our experimental conditions (Figure S4). This discrepancy may be due to differences in the efficiency of USP19 depletion and/or in the proteasomal inhibitor (epoxomicin vs. MG132).

## 4. Materials and Methods

### 4.1. Plasmid Construction

The TM USP19 and FLAG-MARCH6 expression plasmids were described previously [13]. HRD1-FLAG was constructed by cloning a cDNA fragment encoding human HRD1 into the HindIII/XbaI sites of p3× FLAGCMV-14 (Sigma-Aldrich, St. Louis, MO, USA). HRD1^C315S^-FLAG was generated by PCR-based site-directed mutagenesis. FLAG-Nixin was constructed by cloning a cDNA fragment encoding human Nixin into the EcoRV site of p3× FLAGCMV-10 (Sigma-Aldrich). FLAG-HMGCR was constructed by cloning a cDNA fragment encoding human HMGCR into the EcoRI/XbaI sites of p3× FLAGCMV-10 (Sigma-Aldrich). The sequences of all of the plasmids were verified by DNA sequencing.

### 4.2. Antibodies

The following polyclonal and monoclonal antibodies were purchased: anti-USP19 antibody (A301-587A; Bethyl Laboratories, Montgomery, TX, USA); anti-HRD1 antibody (Novus Biologicals, Littleton, CO, USA); anti-α-tubulin and anti-FLAG M2 antibodies (Sigma-Aldrich); anti-c-Myc ant-HA antibodies (Roche, Indianapolis, IN, USA); anti-neomycin phosphotransferase II antibody (NPT II; clone AC113; Merck Millipore, Billerica, MA, USA); anti-K48-linked ubiquitin antibodies (clone Apu2; Merck Millipore); anti-syntaxin 6 antibody (BD Transduction Laboratories, San Jose, CA, USA) and anti-MHC class I antibody (clone EMR8-5; Hokudo, Sapporo, Japan). The anti-USP19 peptide antibody (#1) was made in a rabbit against the synthetic peptides KIDSSNREQRLED and VFYPLVSQSRWR (Operon Biotechnology, Tokyo, Japan) and was used in immunofluorescence staining (Figure S1).

### 4.3. Cell Culture and Plasmid Transfection

The 293T and COS7 cells were cultured in Dulbecco’s modified Eagle medium (DMEM; Sigma-Aldrich) supplemented with 10% fetal bovine serum (FBS), 100 U/mL penicillin, and 100 μg/mL streptomycin at 37 °C in 5% CO_2_. Plasmid transfection was performed with the TransFectin reagent (Bio-Rad, Hercules, CA, USA) according to the manufacturer’s instructions. Imunofluorscence microscopy was performed as described previously [29]. The dilutions of the primary antibodies were: anti-FLAG M2 antibody (1:2000) and anti-USP19 #1 antibody (1:1000).

### 4.4. RNA Interference

Small interference RNA (siRNA)-mediated knockdown of USP19 was performed as described previously [13].

### 4.5. Western Blotting and Immunoprecipitation

Cells were homogenized in TNE buffer (50 mM Tris-HCl, pH 7.4, 150 mM NaCl, 1% Nonidet P-40, 10% glycerol, 1 mM EDTA, 10 mM leupeptin, 1 mM pepstatin, 5 mg/mL aprotinin, and 1 mM phenylmethylsulfonyl fluoride) containing 1 M urea, 10 μM *N*-ethylmaleimide, 2 μM epoxomicin. After the homogenates were then centrifuged at 13,000× *g* for 30 min at 4 °C, the supernatants were analyzed by Western blotting and immunoprecipitation, as described previously [13].

### 4.6. Pulse-Chase Experiments

Pulse-chase experiments were performed as described previously [13]. In brief, cells were labeled for 30 min at 37 °C in cysteine/methionine-free DMEM supplemented with 5% dialyzed FBS and 0.1 mCi/mL (^35^S)cysteine/(^35^S)methionine (PerkinElmer, Waltham, MA, USA). After washing with ice-cold PBS, cells were chased in normal culture medium at 37 °C. Cells were lysed in TNE buffer and then subjected to immunoprecipitation with anti-FLAG M2 antibody beads (Sigma-Aldrich). Immunoprecipitates were analyzed by SDS–PAGE and autoradiography using a FLA7000 phosphorimager (Fujifilm, Tokyo, Japan).

### 4.7. Reverse Transcriptase (RT)-PCR

Total RNAs were extracted from cells with ISOGEN II (Nippon Gene, Tokyo, Japan) and reverse transcribed using ReverTra Ace (Toyobo, Osaka, Japan). RT-PCR was performed with GoTaq Green master mix (Promega, Fitchburg, WI, USA) and the following XBP1-specific primer set: 5′-ttacgagagaaaactcatggcc-3′ and 5′-gggtccaagttgtccagaatgc-3′.

### 4.8. Statistical Analysis

Data are represented as the mean ± SEM of at least three independent experiments. Student’s *t* test or one-way ANOVA followed by Tukey’s *post-hoc* test were used to determine statistical significance. A value of *p* < 0.05 was considered significant.

## 5. Conclusions

In conclusion, we have identified HRD1 as a novel substrate for USP19. USP19 negatively regulates the ubiquitination of HRD1 and prevents it from undergoing proteasomal degradation. These findings will help provide a better understanding of the regulatory mechanisms underlying the ubiquitinating and deubiquitinating enzymes of the ER.

## Supplementary Materials

Supplementary materials can be found at www.mdpi.com/1422-0067/17/11/1829/s1.
