# Supplementary material for: USP19-Mediated Deubiquitination Facilitates the Stabilization of HRD1 Ubiquitin Ligase
Source: Int J Mol Sci. 2016 Nov 2;17(11):1829. doi: 10.3390/ijms17111829 (PMC5133830; doi:10.3390/ijms17111829)
Supplement: Supplementary file 1 [file ijms-17-01829-s001.pdf]

Supplementary Materials: USP19-Mediated Deubiquitination Facilitates the Stabilization of HRD1 Ubiquitin Ligase

Kumi Harada, Masako Kato and Nobuhiro Nakamura

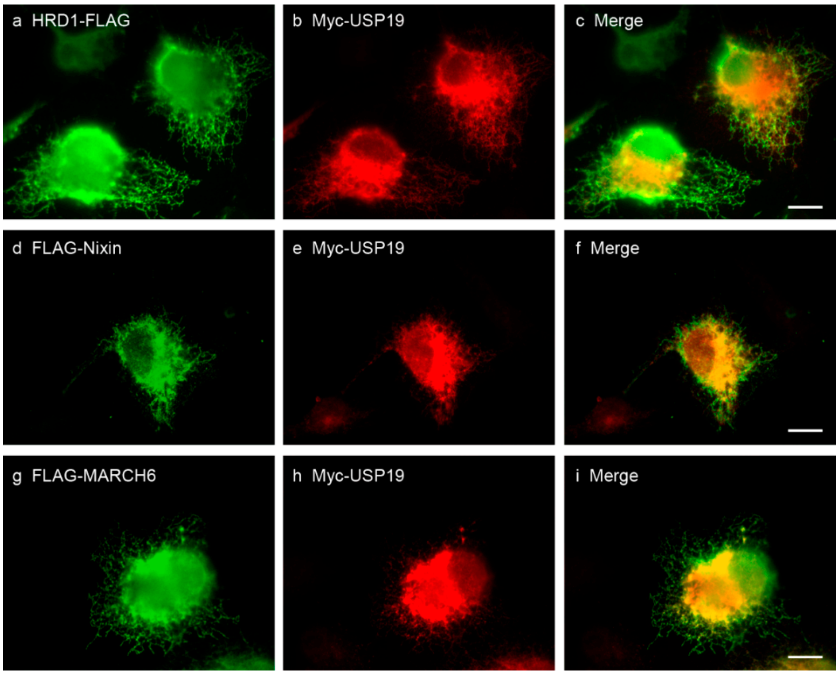

Figure S1. Co-localization of USP19 with ER-localized ubiquitin ligases. COS7 cells were transfected with Myc-USP19 along with either HRD1-FLAG (**a**–**c**), FLAG-Nixin (**d**–**f**) or FLAG-MARCH6 (**g**–**i**). The cells were stained with antibodies against FLAG (green) and USP19 (red). The signals were observed under fluorescence microscopy. Bars, 10 μm.

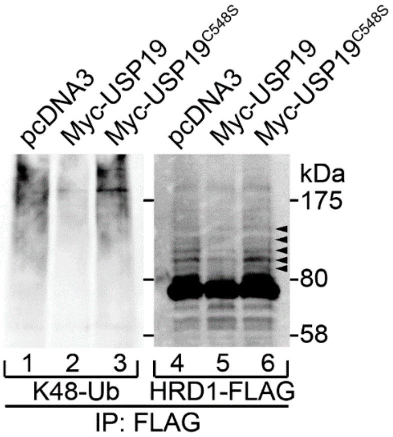

Figure S2. The brightness and contrast of the FLAG blot in Figure 2A were adjusted to make the bands of ubiquitinated HRD1-FLAG (arrowheads) more visible.

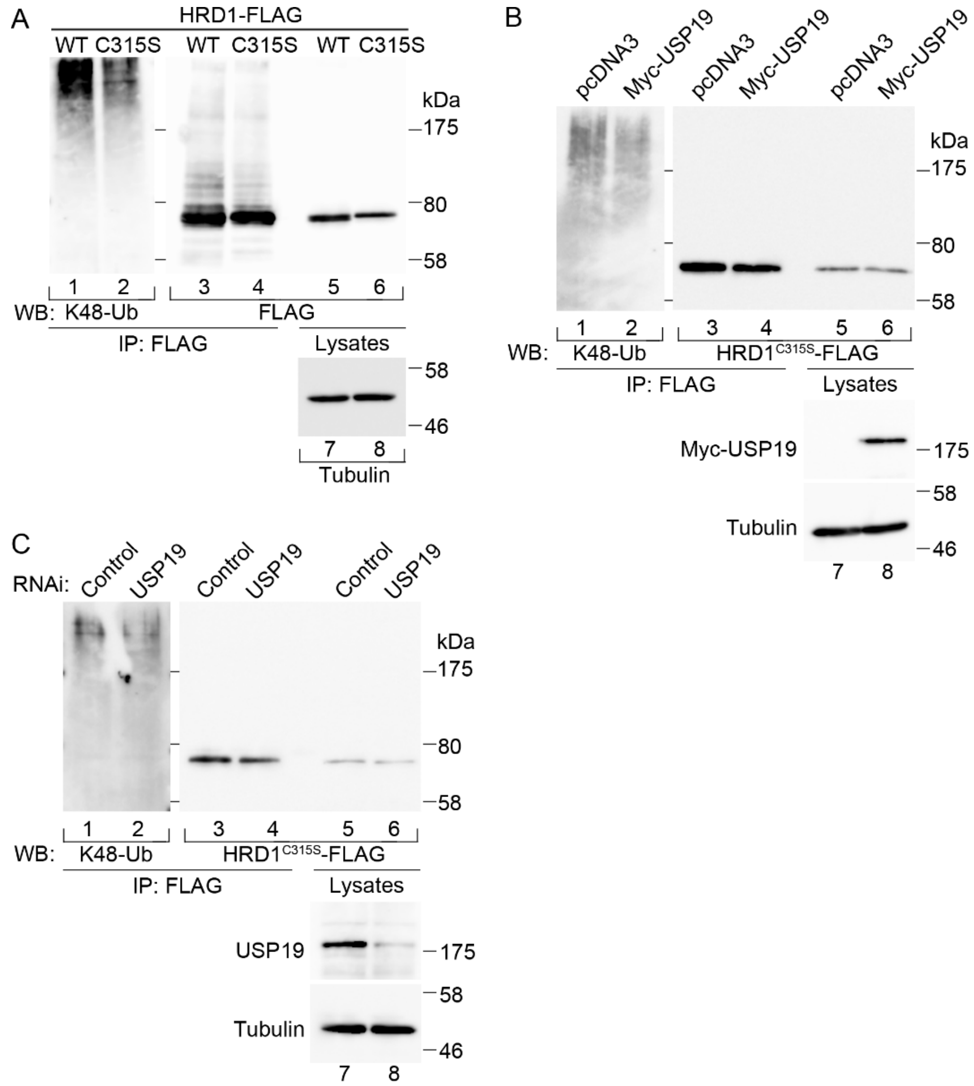

Figure S3. Both overexpression and knockdown of USP19 do not affect K48-linked ubiquitination of HRD1^{C315S}-FLAG. (**A**) Confirmation of autoubiquitination of HRD1. HRD1-FLAG (WT; lanes 1, 3, 5 and 7) and its C315S mutant (C315S; lanes 2, 4, 6 and 8) were transfected into 293T cells After epoxomicin treatment for 6 h, the cells were lysed and then subjected to immunoprecipitation (IP) with an anti-FLAG antibody under denaturing conditions. The immunoprecipitates (50% of the eluates) were analyzed by Western blotting (WB) with antibodies against K48-linked ubiquitin (lanes 1 and 2) and FLAG (lanes 3 and 4). The lysates (20 μg of protein; lanes 5–8) were analyzed by Western blotting with antibodies against FLAG (lanes 5 and 6) and α-tubulin (lanes 7 and 8); (**B**) HRD1^{C315S}-FLAG was transfected into 293T cells along with either pcDNA3 (lanes 1, 3, 5 and 7) or Myc-USP19 (lanes 2, 4, 6 and 8). After epoxomicin treatment, the cells were lysed and then subjected to immunoprecipitation (IP) with an anti-FLAG antibody under denaturing conditions. The immunoprecipitates were analyzed by Western blotting (WB) with antibodies against K48-linked ubiquitin (lanes 1 and 2) and FLAG (lanes 3 and 4). The lysates (20 μg of protein; lanes 5–8) were analyzed by Western blotting with antibodies against FLAG (**top** panel), Myc (**middle** panel) and α-tubulin (**bottom** panel); (**C**) 293T cells were transfected with control siRNA (lanes 1, 3 and 5) or USP19-specific siRNA duplexes (lanes 2, 4 and 6). Two days after transfection, the cells were further transfected with HRD1^{C315S}-FLAG. After epoxomicin treatment, the cells were lysed and then subjected to immunoprecipitation (IP) with an anti-FLAG antibody under denaturing conditions. The immunoprecipitates were analyzed by Western blotting (WB) with antibodies against K48-linked ubiquitin (lanes 1 and 2) and FLAG (lanes 3 and 4). The lysates (20 μg of protein; lanes 5–8) were analyzed by Western blotting with antibodies against FLAG (**top** panel), USP19 (**middle** panel) and α-tubulin (**bottom** panel).

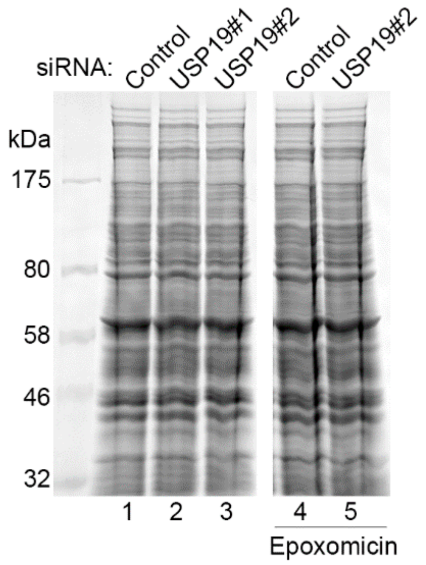

Figure S4. No effect or a lesser effect of USP19 knockdown and proteasomal inhibition on cell viability. The 293T cells were transfected with control siRNA (lanes 1 and 4) or USP19-specific siRNA duplexes (lanes 2, 3 and 5). The cells (lanes 4 and 5) were further transfected with HRD1-FLAG and then treated with epoxomicin for 6 h. Equal volume of cell lysates were separated by SDS–PAGE followed by Coomassie Brilliant Blue staining.

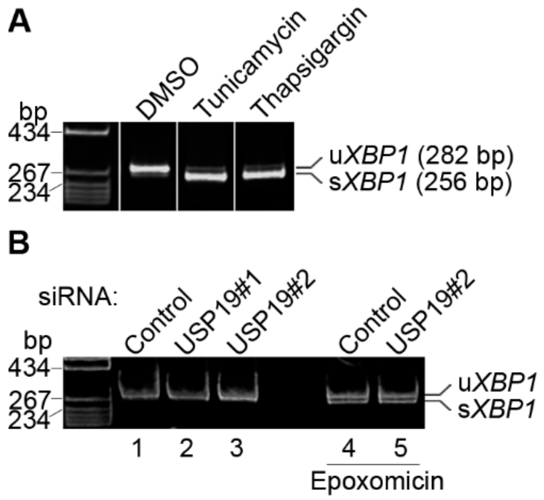

Figure S5. The status of ER stress in USP19-knockeddown cells. (**A**) The 293T cells were treated with vehicle (DMSO), 2 μg/mL tunicamycin or 1 μM thapsigargin for 4 h at 37 °C. RT-PCR was then performed to detect unspliced *XBP1* (u*XBP1*) and spliced *XBP1* (s*XBP1*) transcripts; (**B**) The 293T cells were transfected with control siRNA (lanes 1 and 4) or USP19-specific siRNA duplexes (lanes 2, 3 and 5). The cells (lanes 4 and 5) were further transfected with HRD1-FLAG and then treated with epoxomicin for 6 h. XBP1 transcripts were detected by RT-PCR.

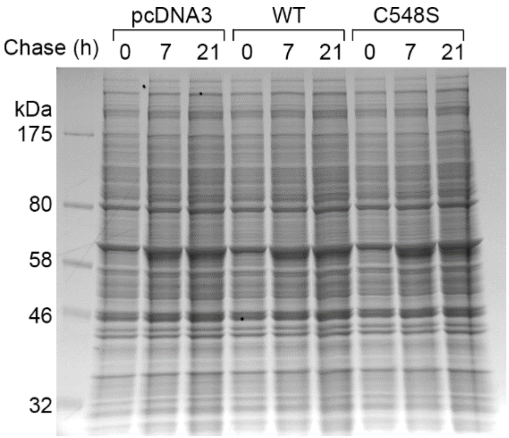

Figure S6. Equal volume of the cell lysates described in Figure 4A were separated by SDS-PAGE followed by Coomassie Brilliant Blue staining.

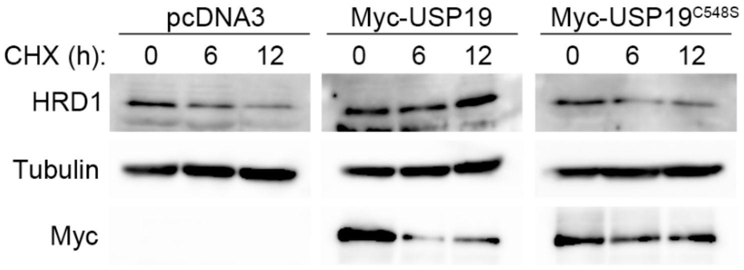

Figure S7. Effect of USP19 activity on the stability of endogenous HRD1. The 293T cells transfected with a pcDNA vector, Myc-USP19 or Myc-USP19^{C548S} were incubated with 0.1 mg/mL cycloheximide (CHX) at 37 °C for indicated time. Whole cell lysates (25 µg of proteins) were analyzed by Western blotting with antibodies against HRD1 (**top** panels), α-tubulin (**middle** panels) and Myc (**bottom** panels).

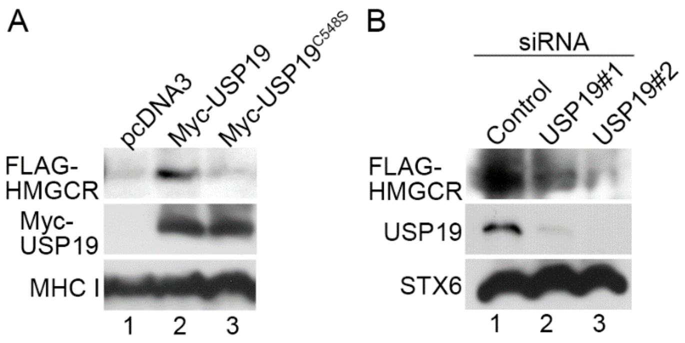

Figure S8. Effects of overexpression and knockdown of USP19 on FLAG-HMGCR expression. (**A**) FLAG-HMGCR was transfected into 293T cells along with either a pcDNA3 vector (lane 1), Myc-USP19 (lane 2) or Myc-USP19^{C548S} (lane 3). Membrane fractions of the cells (60 µg of protein) were analyzed by Western blotting with antibodies against FLAG (**top** panel), Myc (**middle** panel) and MHC class I (as a loading control; **bottom** panel); (**B**) The 293T cells were transfected with control siRNA (lane 1) or USP19-specific siRNA duplexes (lanes 2 and 3). Three days after transfection, the cells were further transfected with FLAG-HMGCR. One day after the second transfection, membrane fractions of the cells (100 µg (**top** panel) and 10 µg of protein (**middle** and **bottom** panels)) were analyzed by Western blotting with antibodies against FLAG (**top** panel), USP19 (**middle** panel) and syntaxin 6 (STX6, as a loading control; **bottom** panel).
